# Characterization of the energy response and backscatter contribution for two electronic personal dosimeter models

**DOI:** 10.1120/jacmp.v16i6.5549

**Published:** 2015-11-08

**Authors:** Joseph Meier, S. Cheenu Kappadath

**Affiliations:** ^1^ Department of Imaging Physics The University of Texas MD Anderson Cancer Center Houston TX USA

**Keywords:** personnel dosimetry, electronic personal dosimeter, radiation protection, nuclear medicine

## Abstract

We characterized the energy response of personal dose equivalent ($H_{p}(10)$ in mrem) and the contribution of backscatter to the readings of two electronic personal dosimeter (EPD) models with radionuclides commonly used in a nuclear medicine clinic. The EPD models characterized were the RADOS RAD‐60R, and the SAIC PD‐10i. The experimental setup and calculation of EPD energy response was based on ANSI/HPS N13.11‐2009. Fifteen RAD‐60R and 2 PD‐10i units were irradiated using ${\;}^{99m}\text{Tc}$, ${\;}^{131}I$, and ${\;}^{131}I$ radionuclides with emission energies at 140 keV, 364 keV, and 511 keV, respectively. At each energy, the EPDs output in $H_{p}(10)$ [mrem] were recorded with 15 inch thick PMMA to simulate backscatter form the torso. Simultaneous free‐in‐air exposure rate measurements were also performed using two Victoreen ionization survey meters to calculate the expected EPD $H_{p}(10)$ values per ANSI/HPS N13.11‐2009. The energy response was calculated by taking the ratio of the EPD $H_{p}(10)$ readings with the expected $H_{p}(10)$ readings and a two‐tailed *z*‐test was used to determine the significance of the ratio deviating away from unity. The contribution from backscatter was calculated by taking the ratio of the EPD $H_{p}(10)$ readings with and without backscatter material. A paired, two‐tailed *t*‐test was used to determine the significance of change in EPD $H_{p}(10)$ readings. The RAD‐60R mean energy response at 140 keV was 0.85, and agreed to within 5% and 11% at 364 and 511 keV, respectively. The PD‐10i mean energy response at 140 keV was 1.20, and agreed to within 5% at 364 and 511 keV, respectively. On average, in the presence of acrylic, RAD‐60R values increased by 32%, 12%, and 14%, at 140, 364, and 511 keV, respectively; all increases were statistically significant. The PD‐10i increased by 25%, 19%, and 10% at 140 keV, 364 keV, and 511 keV, respectively; however, only the 140 keV measurement was statistically significant. Although both EPD models performed within the manufacturers' specifications of ±25% in the energy ranges used, they fell outside of our criteria of 10% at lower energies, suggesting the need to calculate energy‐dependent correction factors, depending on the intended EPD use.

PACS numbers: 87.53.Bn, 87.55.N‐, 87.57.U‐

## I. INTRODUCTION

Electronic personal dosimeters (EPDs) provide the benefit of real‐time dose measurements as a supplement to passive monitoring of whole body radiation dose to radiation workers with thermo luminescent dosimeters (TLD) and optically stimulated luminescent dosimeters (OSLD). EPDs are often used by pregnant technologists in nuclear medicine departments to monitor radiation exposure during pregnancy. The real‐time (e.g., daily) dose measurements provide information that can be used to proactively modify work responsibilities and schedules of the pregnant technologist. This flexibility provides a distinct advantage to relying on monthly readings of whole body dosimeters. EPDs can be used to obtain a better understanding of transient dose received during routine testing of clinical nuclear medicine equipment. The EPDs could also be employed to monitor dose real time during high dose rate procedures, such as administration of therapeutic doses of radionuclides, and to monitor dose to the staff during interventional computed tomography procedures. Even though EPDs are not used as the dose of record but as a dose estimate in these situations, a miscalibrated or faulty EPD could lead to the premature or tardy change of work responsibilities for a pregnant technologist.

EPD vendors quote the energy response to be within 25% across a range of 60 keV to 3 MeV.^1^, ^2^ EPDs are most commonly calibrated with Cs‐137, which has a photon emission at 662 keV. This is higher than typical photon energies that the dosimeters are exposed to most frequently in the nuclear medicine clinic environment, namely 140 keV, 364 keV, and 511 keV from ${\;}^{99m}\text{Tc}$, ${\;}^{131}I$, and ${\;}^{131}I$ radionuclides, respectively. The energy response of the EPDs in this energy range of 140 to 511 keV has not been well characterized, especially using clinically relevant radionuclides commonly used in nuclear medicine clinics.

Other groups^3^, ^4^, ^5^ have used filtered X‐ray spectra close to the energy range in the nuclear medicine clinic, but with dose rates and energy spectra that do not match those encountered in the nuclear medicine clinic. The American National Standards Institute (ANSI) and the Health Physics Society (HPS) have established a standard procedure and criteria for the testing and performance of personnel dosimetry in ANSI/HPS N13.11‐2009.^(6)^

ANSI recommends that polymethyl methacrylate (PMMA) be used to provide backscatter to the EPDs during testing. One group found that backscatter contributed only marginally with an average increase of 2.5% (maximum of 7% for one model) compared to the in‐air EPD reading when irradiated with Cs‐137 for the three models of EPD tested.^(3)^

There are three objectives in this study. The first is to assess the energy response of two EPD models in the energy range experienced in clinical nuclear medicine, and to determine whether energy‐specific energy response correction factors should be applied to individual EPDs. The second objective of this study is to assess the impact of backscatter material on characterizing EPD performance. The third objective is to discuss a practical method for monitoring performance of the EPDs on a periodic basis. We have modified the ANSI/HPS N13.11‐2009 testing procedures of EPDs to make it more suitable for evaluation in a nuclear medicine clinic. We investigated the effects of backscatter on the EPDs motivated by the need to perform periodic testing of EPDs in a simple, clinically relevant manner. If the effect of backscatter can be characterized from comparing irradiations free‐in‐air and irradiations on a phantom, then routine calibrations could be performed free‐in‐air, taking into account the effects of backscatter.

## II. MATERIALS AND METHODS

**Figure 1:**
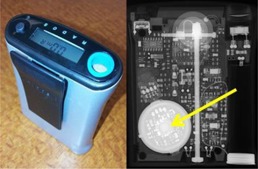
RAD‐60R (left) and radiograph (right). Arrow indicates the PIN Si‐Diode.

**Figure 2:**
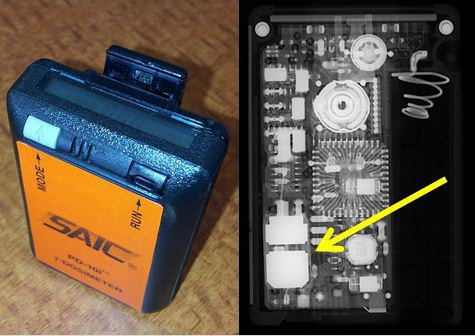
PD‐10i EPD (left) and radiograph (right). Arrow indicates the GM tube.

Two models of EPDs were investigated in this study, the RAD‐60R (RADOS Technology Oy, Turku, Finland) (Fig. 1) and the PD‐10i (Scientific Applications International Corporation, McLean, VA) (Fig. 2). Fifteen RAD‐60R and two PD‐10i units were irradiated using ${\;}^{99m}\text{Tc}$, ${\;}^{131}I$, and ${\;}^{131}I$ radionuclides, corresponding to emission energies at 140 keV, 364 keV, and 511 keV, respectively.

The RAD‐60R EPD uses a $4.5\;{\text{mm}}^{2}$ PIN Si‐Diode, energy compensated with copper filtration.^(1)^ The personal dose equivalent, $H_{p}(10)$, is defined in ICRU Report 51 as the dose equivalent in soft tissue at a depth of 10 mm.^(7)^ This model displays R on the display, but this model actually measures personal dose equivalent, $H_{p}(10)$ in mrem.^(1)^ The specified $H_{p}(10)$ energy dependence is within ±25% for 60 keV to 3 MeV, and within ±35% for 50 keV to 6 MeV. The PIN Si‐Diode is energy compensated with copper filtration.

The PD‐10i employs a Geiger Müller (GM) Tube, energy compensated with aluminum and copper filtration.^(2)^ The specified energy response of that the unit is tissue‐equivalent to within ±25% from 55 keV to 6 MeV. The vendor makes special note that there is a −70% response at 40 keV. This unit display scales automatically between μR, mR, and R. The manual uses dose equivalent (Sv) and exposure (R) interchangeably, stating that 1 Sv=100 R.^(2)^ For the purpose of this characterization and due to its intended use in the clinic, this dosimeter model was assessed under the assumption that the values displayed by the dosimeter are $H_{p}(10)$.

Landauer Inc. specifies the accuracy of their Luxel+ OSLD, used in numerous institutions for tracking exposure to occupational workers, to be within 15% for photons of energy above 20 keV.^(8)^ EPDs are frequently used in situations where more immediate feedback of personnel exposure is necessary. Energy response values of 10% are typically accepted in routine nuclear medicine clinical practice. Therefore, in this study we set the threshold for energy response of 10%, beyond which EPD specific correction factors would be created.

The procedure for characterizing $H_{p}(10)$ for the EPDs was adapted from ANSI/HPS N13.11‐2009 American National Standard for Dosimetry, Personnel Dosimeter Performance — Criteria for testing.^(6)^

ANSI/HPS N13.11‐2009 Section 3.3 provides the suggested radionuclides, particle beams, and ISO 4037‐1 filtered X‐ray spectra that should be used according to which environments the dosimeters will be employed. In our investigations, we used photons of energy 140 keV, 364 keV, and 511 keV from ${\;}^{99m}\text{Tc}$, ${\;}^{131}I$, and ${\;}^{131}I$ radionuclides, respectively. The closest photon sources specified in ANSI compared to those used in this work are Cs‐137 with an emission energy of 662 keV on the high‐energy end, and the filtered X‐ray spectra N200 with an effective energy of 166.3 keV on the low‐energy end. Table 1 shows typical activities and dose rates encountered in the nuclear medicine clinic. The dose rate values are calculated using published values of exposure rate constants.^(9)^

ANSI/HPS N13.11‐2009 Section 3.4 specifies that backscatter shall be provided from PMMA not less than 15 cm deep, and with a face measuring no less than 30 cm×30 cm. All phantoms for backscatter used in our investigations satisfied these criteria.

ANSI/HPS N13.11‐2009 Section 3.5 specifies that the source to sensitive element distance shall be no less than 1 m, and that the sensitive element of the dosimeter shall not be placed closer than 7.5 cm from the lateral edge of the backscatter phantom. For all acquisitions, the sensitive elements of the EPDs and the center of the ionization chambers were placed at 1.2 m from the radioactive source.

ANSI/HPS N13.11‐2009 Section 3.7 specifies that the air‐kerma measurements are to be made in the absence of scatter. Scatter was reduced by positioning the center of the ionization chambers 108 cm above the floor on top of empty cardboard boxes. Two calibrated Victoreen 451‐B (Fluke Biomedical, Everett, WA) ionization chamber survey meters were used to measure the exposure from radionuclides during measurements for the characterizing of $H_{p}(10)$ for the EPDs. The ionization chamber was operated in integration mode to accumulate the total exposure over the measurement time interval. The ionization chamber survey meter vendor reports an accuracy of ±10% of the reading.^(10)^ A relative error of 10% stemming from accuracy of the ion chamber measurements has been incorporated into the total error estimates for the energy response of EPD $H_{p}(10)$. The energy dependence of the ionization chamber accuracy, $C_{x}$, was obtained from the vendor's energy dependence curve and applied to the average exposure readings.^(10)^ Air kerma, $K_{a}$, was calculated by applying the usual conversion from exposure readings, $XM, to air kerma, via 0.87 mrad/mR such that

(1) $$K_{a}=\bar{X}*0.87*\frac{1}{C_{x}}$$

ANSI/HPS N13.11‐2009 specifies that the personal dose equivalent at 10 mm, $H_{p}(10)$, shall be assigned as follows:

(2) $$H_{p}(10)=c_{K,d,\alpha }K_{a}$$

where $K_{a}$ is the measured air kerma in the absence of scatter, and $c_{K,d,\alpha }$ is the energy dependent conversion coefficient of air kerma, $K_{a}$, to $H_{p}(10)$ provided by ICRU Report 57 in its Table A.24.^6^, ^11^ The table of conversion factors provided in ICRU 57 were calculated by via Monte Carlo simulations for monoenergetic photon beams. The conversion factors specific to the energies of interest in this study were calculated via linear interpolation from the table provided in ICRU 57 and are listed in Table 2.

**Table 1:** Typical radionuclides, energies, activities, and dose rate range in the nuclear medicine clinic at 1 m. The dose rate values are calculated from exposure rate constants from Cherry et al.^(9)^

*Radionuclide*	*Half Life (hr)*	*Energy (keV)*	*Activity Range (mCi)*	*Low Dose Rate (mrem/hr)*	*High Dose Rate (mrem/hr)*
Tc‐99m	6.01	140	1‐50	0.12	6.14
I‐131	192.47	346	1‐250	0.28	69.07
F‐18	1.83	511	1‐20	0.69	13.91

**Table 2:** $K_{a}$ to $H_{p}(10)$ values used in this work derived via linear interpolation of values provided by ICRU Report 57, Table A.24. The energy dependence of the ionization chamber accuracy used to correct the ion chamber readings is also reported

*Radionuclide*	*Energy (keV)*	$H_{p}(10)/K_{a}$ *(Sv/Gy)*	*451‐B Energy Correction Factor* $C_{x}$
Tc‐99m	140	1.64	1.08
I‐131	364	1.32	1.03
F‐18	511	1.25	1.01

### A. $H_{p}(10)$ energy response

**Figure 3:**
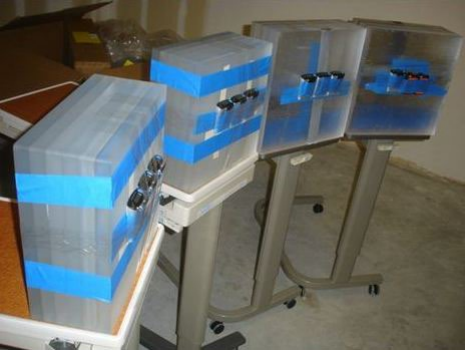
Setup of EPD irradiation with backscatter from PMMA per ANSI/HPS N13.11‐2009. The EPDs are located 120 cm away from the radioactive source and 108 cm above the floor.

**Figure 4:**
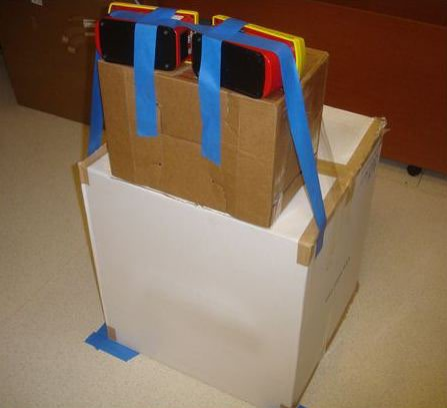
Setup of ionization chambers for exposure‐rate measurements concurrent with EPD irradiation. The ionization chambers are located 120 cm away from the radioactive source and 108 cm above the floor.

**Figure 5:**
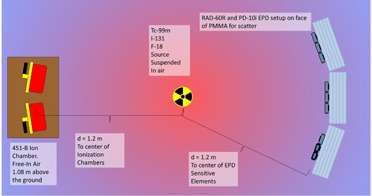
Schematic of the setup for simultaneous irradiation of the EPDs and ionization chambers.

In order to assess the energy response of the EPDs with respect to emissions of varying energies, simultaneous irradiations were made of the EPDs with backscatter (Fig. 3) and of the ionization chambers with no backscatter (Fig. 4) per ANSI/HPS N13.11‐2009. The center of the ionization chambers and the center of the sensitive elements of the EPDs were placed 1.2 m away from the radionuclide of interest (Fig. 5). The centers of the sensitive elements of the EPDs were positioned height‐wise in the center of the PMMA phantoms. The center of the sensitive elements of the ionization chambers, the sensitive elements of the EPDs, and the radionuclide source were placed 108 cm above the floor in an effort to minimize scatter contributions from the concrete floor. We conservatively estimate that setup of ionization chambers and EPDs was within 10 mm of the reported values. An error of ±10 mm would translate via the inverse‐square dependence to an uncertainty of ±2.0% on the ionization chamber and EPD measurements. A relative error of 2% stemming from distance uncertainty has been incorporated into the total error estimates for the energy response of EPD $H_{p}(10)$. Multiple (6 to 10) measurements of energy response were performed for each of the EPDs at each of the different energies. In order to account for radioactive decay during the measurements, the ionization chambers and EPDs were operated in integration mode to accumulate the total exposure over the measurement time interval. Table 3 summarizes the radionuclide exposure rates, acquisition times, number of repeat acquisitions, and other relevant information regarding data acquisition. For each measurement, the expected value of $H_{p}(10)$ was calculated according to Eq. (2). The energy response of each $H_{p}(10)$ measurement for each EPD was calculated by taking the ratio of each of the EPD $H_{p}(10)$ readings to the corresponding ionization chamber values for expected $H_{p}(10)$. The mean energy response for each EPD tested at a given energy was determined by averaging the energy response values over the multiple measurements made at that energy; the standard error for the energy response was also calculated. A two‐tailed z‐test was performed based on the standard error with a threshold value of p=0.05 to identify EPDs whose energy response diverged significantly from unity. The mean energy response and its range were calculated for the population of RAD‐60R and PD‐10i EPDs tested at all energies. The overall uncertainty in energy response for each dosimeter was computed by adding the 2% relative error from distance uncertainty and the 10% relative error from ion chamber accuracy to the standard error in quadrature. The entire group of fifteen RAD‐60R EPDs was characterized with Tc‐99m.

**Table 3:** Summary of data acquisition. Tc‐99m data were acquired on two separate days

*Radionuclide*	*Tc‐99m*	*Tc‐99m*	*I‐131*	*F‐18*
Half Life (hr)	6.01	6.01	192.47	1.83
Beginning dctivity (mCi)	100	105.9	3.86	18
# Acquisitions free‐in‐air	0	8	6	8
# Acquisitions with scatter	10	8	6	8
# EPDs irradiated free‐in‐air	0	17	17	17
# EPDs irradiated with backscatter	6	12	8	8
Initial exposure rate @ 1.2 m (mR/hr)	5.04	5.36	0.63	7.5
Final exposure rate @ 1.2m (mR/hr)	3.38	1.44	0.57	1.5
Initial exposure time (min)	16	15	100	10
Final exposure time (min)	24	55	112	50

However, only six RAD‐60R EPDs were characterized with I‐131 and F‐18 radionuclides. Both of the PD‐10i EPD units were characterized at all energies.

### B. Dependence of EPD energy response on the presence of backscatter

**Figure 6:**
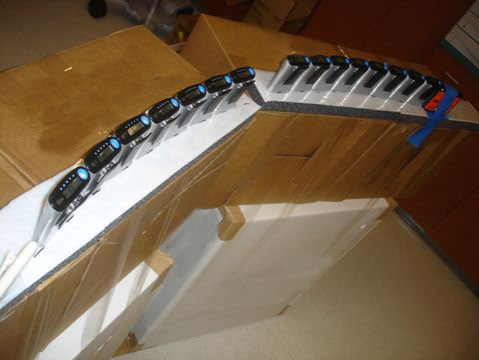
The set‐up for free‐in‐air irradiation of EPDs. The EPDs are located 120 cm away from the radioactive source and 108 cm above the floor.

To assess the impact of backscatter provided by the PMMA on the EPD energy response, the EPDs were irradiated with and without backscatter from PMMA under identical conditions at all energies. The experimental setup for the EPD acquisitions with backscatter was the same as in the Materials & Methods section A above. To irradiate the EPDs without scatter, they were placed on top of cardboard boxes at a distance of 1.2 m from the source, and with the center of the respective sensitive elements placed at a height of 108 cm (Fig. 6). During all acquisitions, the ionization chambers were simultaneously irradiated with the same setup as in the Materials & Methods section A. Table 3 summarizes the data acquisitions.

As in section A above, the expected value of $H_{p}(10)$ for each EPD was calculated for each acquisition. The reading of each EPD for each acquisition was normalized to the expected value of $H_{p}(10)$. The backscatter factor, defined as the ratio of the mean normalized readings with backscatter to the mean normalized readings without backscatter, was calculated for each EPD unit. In addition, the mean and range for backscatter factor were determined for the population of RAD‐60R and PD‐10i for all energies. A paired, two tailed *t*‐test with a threshold value of p=0.05 was performed for each dosimeter model to assess differences in the mean readings of the each EPD model population with and without backscatter at each energy.

## III. RESULTS

### A. $H_{p}(10)$ energy response

At all energies for both EPD models, the energy response of the EPDs for both models did not diverge from unity by greater than 25%, and thus all were operating within the manufacturers' energy response specifications of ±25% for energies between 60 keV and 3 MeV.

At 140 keV, the 15 RAD‐60R EPDs had a mean energy response of 0.85, with a minimum value of 0.76 and a maximum value of 0.96; 14 units were significantly different from 1.0 (p<0.05), and 13 had energy response values that diverged by more than 10% from unity. At 364 keV, the six RAD‐60R EPDs tested had a mean energy response of 1.03, with a minimum value of 1.00 and a maximum value of 1.07; none of the units were significantly different from 1.0 (p>0.05), and none had energy response values that diverged by more than 10% from unity. At 511 keV, the six RAD‐60R EPDs tested had a mean energy response of 1.11, with a minimum value of 1.08 and a maximum value of 1.17; three of the units were significantly different from 1.0 (p<0.05), and three had energy response values that diverged by more than 10% from unity.

At 140 keV, the two PD‐10i EPDs had a mean energy response of 1.20, with a minimum value of 1.18 and a maximum value of 1.21; both units were significantly different from 1.0 (p<0.05), and both had energy response values that diverged by more than 10% from unity. At 364 keV, the two PD‐10i EPDs had a mean energy response of 1.04, with a minimum value of 1.03 and a maximum value of 1.06; one unit was significantly different from 1.0 (p<0.05), and neither had energy response values that diverged by more than 10% from unity. At 511 keV, the two PD‐10i EPDs had a mean energy response of 1.04, with a minimum value of 1.02 and a maximum value of 1.06; one unit was significantly different from 1.0 (p<0.05), and neither had energy response values that diverged by more than 10% from unity.

**Figure 7:**
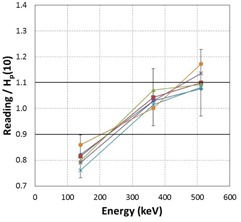
The observed energy response of six RAD‐60R evaluated together with their overall 1‐sigma uncertainty as a function of photon energy. Errors bars for all dosimeters are of similar magnitude, but have been plotted for one EPD only, for clarity.

**Figure 8:**
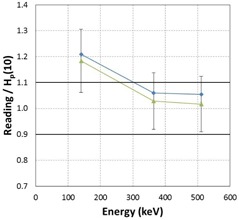
The observed energy response of two PD‐10i evaluated together with their overall 1‐sigma uncertainty as a function of photon energy. Errors bars for both dosimeters are of similar magnitude but have been plotted for one EPD only, for clarity.

**Figure 9:**
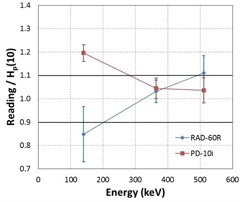
The average energy response for the RAD‐60R and PD‐10i dosimeter models. The error bars represent the 95% CIs.

The calculated energy response values for the EPDs investigated are summarized in Table 4. Figures 7 and 8 show the energy response of the EPDs together with their overall 1‐sigma uncertainty as a function of photon energy for the six RAD‐60R and two PD‐10i EPDs, respectively. The mean overall uncertainty for all EPDs tested at all energies was calculated as 10.7% with a maximum of 13.6%. A plot of the average energy response for each EPD model along with their 95% confidence limits versus photon energy is shown in Fig. 9.

**Table 4:** The mean, minimum, and maximum $H_{p}(10)$ energy response for the population of two EPD models investigated. Also tabulated are the fractions of dosimeters whose measured $H_{p}(10)$ energy response (μ) deviated by greater than 10% from unity and the fraction of dosimeters with a p‐value <0.05 for a two‐tailed *z*‐test

	*RAD‐60R*						*PD‐10i*					
*Energy (keV)*	# *EPDs*	*Mean*	*Min*	*Max*	*#EPDs* 0.9<μ<1.1	*Two‐tail z‐test* p<0.05	# *EPDs*	*Mean*	*Min*	*Max*	*#EPDs* 0.9<μ<1.1	*Two‐tail z‐test* p<0.05
140	15	0.85	0.76	0.96	2/15	14/15	*2*	1.20	1.18	1.21	2/2	2/2
364	6	1.03	1.00	1.07	6/6	0/6	*2*	1.04	1.03	1.06	0/2	1/2
511	6	1.11	1.08	1.17	3/6	3/6	*2*	1.04	1.02	1.06	0/2	1/2

### B. Dependence of energy response on the presence of backscatter

At all energies, the mean backscatter factor for either EPD model was greater than or equal to 1.10, implying that the presence of backscatter material increased the EPD measurement by greater than or equal to 10%. At 140 keV, the 10 RAD‐60R EPDs had a mean backscatter factor of 1.32 (significantly different than 1.0 with p<0.001), with a minimum value of 1.23 and a maximum value of 1.37. At 364 keV, the six RAD‐60R EPDs had a mean backscatter factor of 1.12 (significantly different than 1.0 with p<0.003), with a minimum value of 1.06 and a maximum value of 1.23. At 511 keV, the six RAD‐60R EPDs had a mean backscatter factor of 1.14 (significantly different than 1.0 with p<0.003), with a minimum value of 1.05 and a maximum value of 1.21.

At 140 keV, the two PD‐10i EPDs had a mean backscatter factor of 1.25 (marginally different than 1.0 with a p‐value of 0.03, with a minimum value of 1.23 and a maximum value of 1.27. At 364 keV, the two PD‐10i EPDs had a mean backscatter factor of 1.19 (not statistically different from unity), with a minimum value of 1.14 and a maximum value of 1.24. At 511 keV, the two PD‐10i EPDs had a mean backscatter factor of 1.10 (not statistically different from unity), with a minimum value of 1.06 and a maximum value of 1.15.

**Figure 10:**
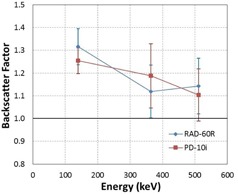
The average backscatter factor for the RAD‐60R and PD‐10i dosimeter models. The error bars represent the 95%CIs.

The backscatter factors for both EPD models are summarized in Table 5. The plots of average backscatter factors for both EPD models as a function of energy are shown in Fig. 10, along with their 95% confidence limits.

**Table 5:** The backscatter factors for the two EPD models investigated. The significance threshold p‐value for the paired two‐tailed *t*‐test is 0.05

	*RAD‐60R*					*PD‐10i*				
*Energy (keV)*	*#EPDs*	*Mean*	*Min*	*Max*	*Paired t‐test p‐value*	*#EPDs*	*Mean*	*Min*	*Max*	*Paired t‐test p‐value*
140	10	1.32	1.23	1.37	p<0.01	2	1.25	1.23	1.27	0.03
364	6	1.12	1.06	1.23	p<0.01	2	1.19	1.14	1.24	0.15
511	6	1.14	1.05	1.21	p<0.01	2	1.10	1.06	1.15	0.22

## IV. DISCUSSION

### A. $H_{p}(10)$ energy dependency

Characterizations of EPDs have been conducted by others, and they have shown that there is a very broad performance in the energy response of the many EPDs that are available on the market with respect to radiation type, photon energy, photon beam quality, and the dose rate.^3^, ^4^, ^5^ Although all evaluated EPDs operated within the manufacturers' energy response specifications of ±25%, our results show that both of the EPD models investigated exhibited an energy‐dependent response, at least between 140 to 511 keV. At 140 keV, the energy response of both EPD models diverged on average by greater than 10% from unity; the RAD‐60R under‐responded by 15%, whereas PD‐10i over‐responded at 20% (Fig. 9). The majority of the EDPs did not meet our energy response expectation of 10% accuracy at 140 keV. The average energy response of the EPDs trends toward unity as the photon energy increased to 662 keV, the energy at which the EPDs were initially calibrated (Fig. 9). Even though this trend might suggest that corrections are not needed at higher energies, at 511 keV, three of the six RAD‐60R EPDs had energy response values that were greater than 10%, the highest being 1.17. The range of energy response values that was observed in the EPDs tested, and the range of performance that has been shown by others, suggest the need to characterize the performance appropriate to the intended use of the EPDs prior to implementation. Therefore, we recommend that users consider the need to establish EPD‐specific correction factors prior to clinical use, at least at 140 keV. The EPD‐specific correction factors are calculated by taking the inverse of the EPD energy response (see Results section A) at each energy. The correction factor is utilized by taking the product of the actual EPD reading and the EPD‐specific correction factor as the corrected $H_{p}(10)$ value in mrem.

The energy response of EPD $H_{p}(10)$ readings was assessed multiple times as the radionuclide decayed; the initial and final exposure rates are reported in Table 3. We analyzed the resultant $H_{p}(10)$ values and determined that there was no statistically significant dependence on dose rates, at least at dose rates present in the nuclear medicine clinic. The mean overall uncertainty in $H_{p}(10)$ values for all EPDs tested at all energies, that includes the uncertainty contributions from ionization chamber survey meter readings (∼10%), distance errors (∼2%), and random errors in measurements (∼3%), was calculated as 10.7% with a maximum of 13.6%.

At 140 keV, the average response of the RAD‐60R is 0.85, while that for the PD‐10i is 1.20. This difference in energy response between the two models could possibly be explained by noting the difference in the detecting element of the two models — the RAD‐60R using a PIN Silicon Diode energy compensated with copper filtration, and the PD‐10i using a GM tube energy compensated with aluminum and copper filtration. The PD‐10i converts each count from the GM tube into dose via a linear calibration factor.^(2)^ A GM tube treats any interaction of any photon energy that is sufficient to induce an avalanche in the sensitive volume as one count. This linear conversion factor which converts one count into a corresponding dose is only appropriate for energies in the range of the calibration source. As the photon energies decrease from 662 keV, it then follows that the energy response of the PD‐10i would increase above unity (as observed in our measurements). The PD‐10i manufacturer also states that, at 40 keV, there is a −70% response. This could be explained by the fact that the energy compensation copper and aluminum filtration surrounding the GM tube attenuates a significant portion of the 40 keV and lower energy photons. It is expected for the RAD‐60R that the PIN diode would decrease in energy response as the energy of the photos decreases from the calibration energy of 662 keV (as observed in our measurements). Interactions inside of the PIN diode create signal that is proportional to the energy deposited, in contrast to the GM tube in which the same amount of signal is produced with any amount of energy deposited from an individual event. For the RAD‐60R, as the photon energy decreases, the copper filtration surrounding the PIN diode attenuates more of the incoming photons, thus causing the decrease in energy response, which is reflected in our data.

### B. Assessment of the necessity for providing backscatter during calibrations

Our results showed, across all energies tested, that the mean backscatter factor is greater than or equal to 1.10, and that the range of the average backscatter factor ranged between 1.10 and 1.32. Given an energy response expectation of ±10%, our data show that in the energy range relevant to nuclear medicine clinics from 140 keV to 511 keV, it is necessary to perform energy response characterizations that include backscatter from PMMA. As expected, the backscatter factor decreased with increasing photon energy (Fig. 10) because Compton‐scattered photons at higher energies are distributed preferentially in the forward direction.

### C. Periodic testing and EPD‐specific correction factors

The characterization methods aforementioned are labor‐intensive and time‐consuming due primarily to the fact that in our case we used unsealed radionuclides of relatively low activity, and secondly because it is very laborious to set up four 15 inch PMMA slabs to perform simultaneous irradiations of all of the EPDs with PMMA. Due to the irradiation distances that ANSI/HPS specifies, the dose rate in our case is relatively low and the acquisition lengths are relatively long, in comparison with the rates and times used by others who used filtered X‐ray beams and Cs‐137 and Co‐60 irradiation vaults with much higher dose rates and much shorter acquisition lengths. To avoid the long acquisition times and setup of the acquisitions with PMMA, routine testing and in‐house, EPD‐specific correction factors could be carried out in the absence of scatter. The readings of the EPDs irradiated in the absence of scatter would be multiplied by the user‐determined, EPD‐specific backscatter factors to provide a value equal to that which would be read in the presence of scatter. These normalized readings could then be compared to the true value of $H_{p}(10)$, measured with the procedure outlined in the Material & Methods section A.

After the initial energy‐dependent assessment of energy response of EPDs, further simplification of periodic testing could be achieved by foregoing the direct measurement of $H_{p}(10)$ with ionization chambers, but rather focus on relative change in performance over time. The EPDs could be tested at much closer irradiation distances (higher dose rates) — for example, locate the entire EPD population to be tested in a ring around a NIST traceable Cs‐137 standard that nuclear medicine clinics routinely utilizes for dose calibrator constancy. The user would then determine the ratio of the EPD reading to the activity of the standard, as labeled on the radioactive source. Provided that the same irradiation conditions are used, and by accounting for source decay, the EPD constancy could be easily assessed on a yearly or more frequent basis.

## V. CONCLUSIONS

The RAD‐60R and the PD‐10i units have energy response values that significantly diverge from unity at 140 keV, but trend toward unity as the incident photon energy increases to 511 keV. In the energy ranges investigated in this study, both EPD models were shown to have significant increases in reading in the presence of backscatter from PMMA. Our data demonstrate that, if these EPD models are to be used as the dose of record or to be used to preemptively change worker assignments, then testing and calibrations of EPD specific to the environment of deployment is a prerequisite. We recommend that the user perform periodic testing that is commensurate with the intended application of the EPDs.
